# Measuring a Lagrangian drifter’s slip with an onboard ADCP

**DOI:** 10.1016/j.mex.2019.05.032

**Published:** 2019-06-01

**Authors:** J.L. Herrera, J. González, R.A. Varela

**Affiliations:** a Grupo de Oceanografía Física, Universidad de Vigo, Facultad de C.C. del Mar, Campus de Vigo, 36310 Vigo, Spain; b Estación de Ciencias Mariñas de Toralla, Universidad de Vigo, Illa de Toralla s/n, 36331 Vigo, Spain

**Keywords:** In-situ drifter slip estimation, Drifter performance, Lagrangian current profiles, Heavily instrumented drifter

## Abstract

A perfect Lagrangian drifter should move with the same velocity as the water volume that it is following. Deviations from this ideal will result in a relative velocity between the drifter’s drogue and its surrounding water, commonly named “slip”. Estimating a drifter’s slip is difficult, especially for custom and heavily instrumented drifters. We propose to use a Self-Contained Acoustic Doppler Current Profiler (SCADCP) attached to the drifter to:

• Measure the drifter’s slip directly at the drogue depth.
• Obtain complementary data of current at other depths.

## Graphical abstract

**Figure:**
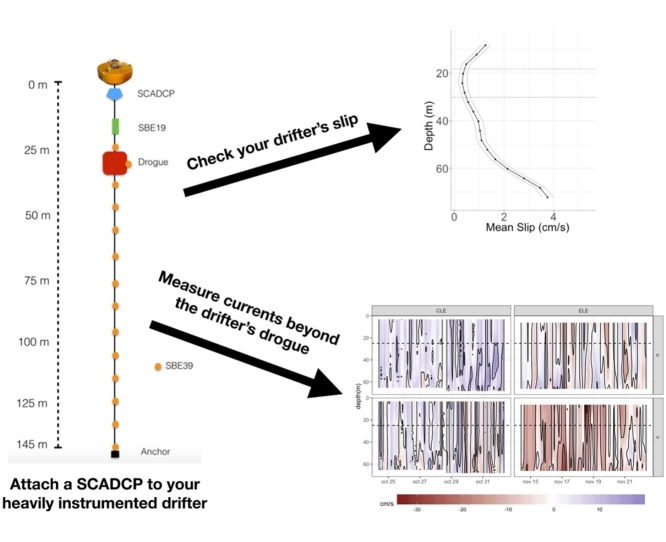

**Specifications Table**

**Table:** 

Subject area:	*Earth and Planetary Sciences*
More specific subject area:	*Physical Oceanography*
Method name:	*In-situ drifter slip estimation*
Name and reference of original method:	*N/A*
Resource availability:	*N/A*

## Method details

Lagrangian current velocity data derived from observations of floating objects’ motion are extremely valuable, but the success of a Lagrangian experiment relies on the performance of the drifter used. Ideally, the speed of the drifter relative to the water volume located at the drifter’s drogue depth should be very close to zero. Deviations from that will result in relative velocity between the drifter’s drogue and its surrounding water, commonly named “slip”.

Often, in-situ estimation of a drifter’s slip is the only practical way to determine a drifter’s performance. That is especially true when we deal with custom and heavily instrumented drifters. An approach used for the estimation of a drifter’s slip is to obtain direct measurements of the slip. Niiler et al. [1] and O’Donnell et al. [2] attached current meters above and below the drogue of a TRISTAR-I and FGGE drifter to measure their slip. Nevertheless, these observations were restricted to the depths where the current meters were attached, and uncertainty arose when the drogue was located at depths with strong vertical velocity shears.

In this paper, we propose to use a Self-Contained Acoustic Doppler Current Profiler (SCADCP) attached to the drifter to measure the slip directly at the drogue depth. The current velocity profiles recorded by a SCADCP attached to the drifter are relative to the drifter movement. Here, the drifter plays the role of a moving reference frame. If the drogue performance is perfect, the SCADCP would theoretically record zero speed at the drogue depth (zero slip), or in practice, values under the instrument's error.

To get a measure of the slip at the drogue depth, we may want to consider the following when we design our experiment:

1 The drogue depth should be within the SCADCP measuring range, the closer to the SCADCP, the better; as SCADCP measurement error increases with distance.
2 The bin size should be small enough to locate the position of the drogue at every current profile.

One fundamental advantage of the ADCP technology is that the measured currents are not restricted to the location of the instrument, but reach up to a distance determined by the ADCP design and configuration. Therefore, a SCADCP attached to the drifter also provides with complementary data of currents at depths others than the drogue depth. Those current profiles can be used as input in other applications like computing vertical shear. As the SCADCP is attached to the drifter, when the velocity of the drifter (the velocity of the moving reference frame) is added to the SCADCP profiles, we obtain absolute current profiles relative to the same reference frame used to measure the drifter speed – usually a geographic reference frame.

### Method limitations

Some situations could increase slip uncertainty. In rough seas, the buoy will be unstable and transfer its movement to the SCADCP. The SCADCP will register the buoy accelerations, which in turn will bias the slip estimate. Setting an ensemble period long enough to average out the buoy movement could address the problem; however, the uncertainty is likely to increase.

In other cases, a strong vertical gradient along the drogue will make it harder to estimate the slip. One approach is to average the slip through the drogue depth range. However, slip uncertainty will increase nonetheless.

### Validation

**Fig. 1:**
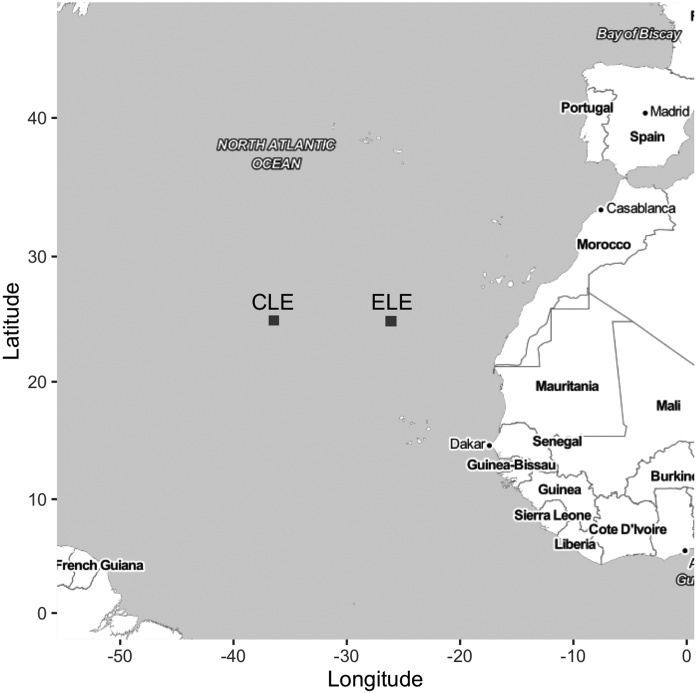
Locations of the Central Lagrangian Experiment (CLE) and the Eastern Lagrangian Experiment (ELE).

To illustrate our method, we will use two Lagrangian experiments performed in the frame of the CARPOS project (in Spanish “flujos de CARbono mediados por el Plancton en ambientes Oligotróficos Subtropicales” or “plankton-mediated carbon fluxes in contrasting subtropical oligo- trophic environments: a Lagrangian approach” in english) funded by the Spanish government (MEC, REN2003-09532- C03). One experiment was named center Lagrangian experiment (CLE) and was located at the center of the North Atlantic Subtropical Gyre (in the neighborhood of 36.5 °W 25 °N, Fig. 1). The other, named eastern Lagrangian experiment (ELE), was performed at the eastern margin of the Gyre (close to 26 °W 25 °N, Fig. 1).

CARPOS aimed to study the carbon fluxes that take place in the North Atlantic subtropical gyre pelagic ecosystem [3]. One particular objective of the project was to investigate the short time variability of the biogeochemical balances in two moving volumes of water. During each Lagrangian experiment, a heavily instrumented custom drifter was tracked to sample the biogeochemical and physical properties of its surrounding water intensively. The performance of the Lagrangian drifter conditioned the ability to distinguish the geographical from the temporal variability of the biogeochemical balances. The lack of previous information about the drifter behavior and its critical role in the project motivated the development of a system exclusively devoted to measuring the drifter slip.

**Fig. 2:**
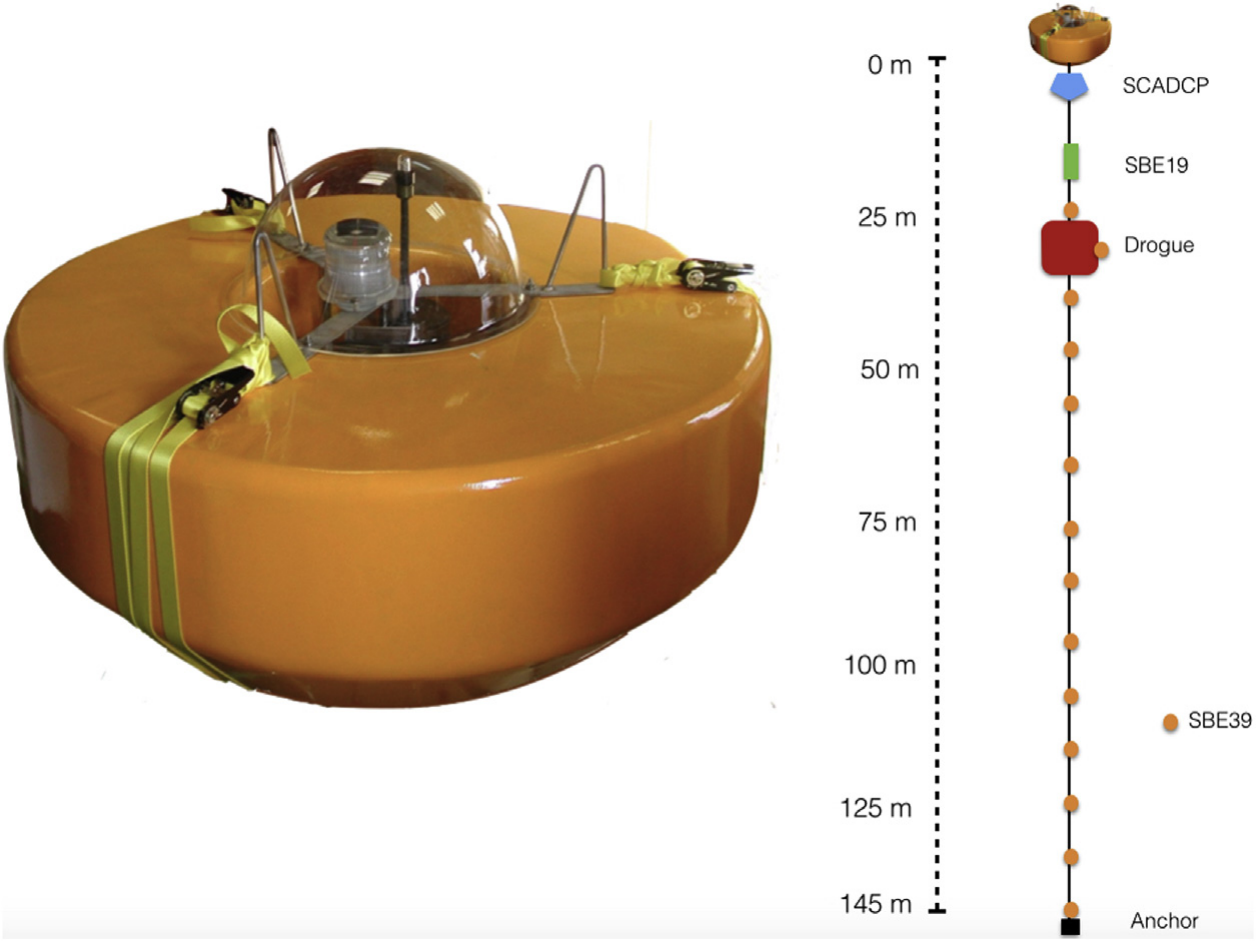
View of the buoy used in the Lagrangian experiments in the left side, where can be observed an inner compartment where a flashlight, antennas, and communication system were allocated. In the right, representation of the vertical distribution of the drifter deployed; under the buoy are installed from up to down, the SCADCP, a CTD (SBE19) and the drogue, with fourteen sensors for temperature and pressure distributed along the tether (SBE39).

Typical designs for relatively inexpensive mixed-layer drifters share three features: navigation and data telemetry package; a drag element or drogue; and housing for electronics, power packs, and antennas included in a surface buoy or float [2]. The configuration of the CARPOS drifter followed this model and was the same in both experiments (Fig. 2). We describe the drifter design in greater detail in the Supplementary Materials section.

A downward-looking RDI 300 kHz Workhorse SCADCP was inserted inline in the tether connecting the surface buoy and the drogue, and at 2 m depth below the surface buoy. It was configured to sample currents in 1 m bins beginning at 5.24 m depth. SCADCP data were inspected after CLE, and it was determined that such a high vertical resolution was not needed. Thus, currents were sampled into 4 m bins beginning at 8.22 m from the surface during ELE. This modification of the vertical resolution allowed to decrease the instrumental error (standard deviation decreased from 2.29 cms-1 to 0.70 cms-1), also reducing the pinging rate of the instrument for battery saving purposes. Although the ensemble averaging period of the SCADCP was set to 1 min in both experiments, the instrument pinging rate was set to 35 pings/minute during CLE and 26 pings/minute during ELE.

**Fig. 3:**
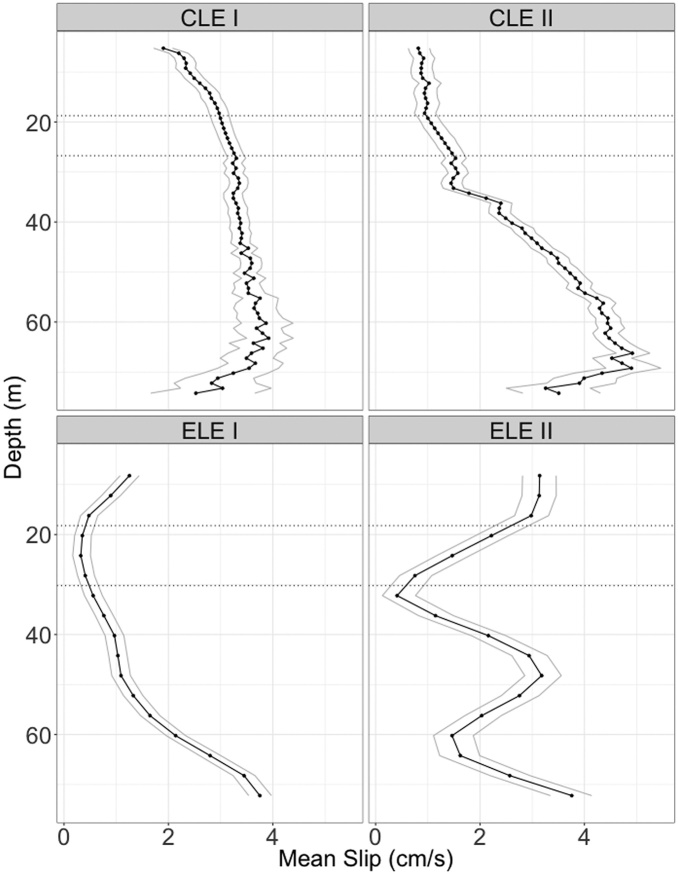
Mean speed measured by the SCADCP moored below the drifter’s buoy (solid black line) during a) the first and b) the second part of CLE and for c) the first and d) second part of ELE. Grey lines show 95% confidence interval. The horizontal dotted lines delimit the drogue depth range. Dots correspond to the bin resolution.

The drifter's drogue interfered the SCADCP beams and we discarded all the data at the depth range occupied by the drogue: from 19 to 26 m (8 bins) during CLE and from 20 m to 28 m (3 bins) during ELE. Fortunately, having current profiles instead of currents at a limited number of discrete points provides enough information to interpolate the data corresponding to those depth ranges (Fig. 3) using a spline algorithm [4].

**Fig. 4:**
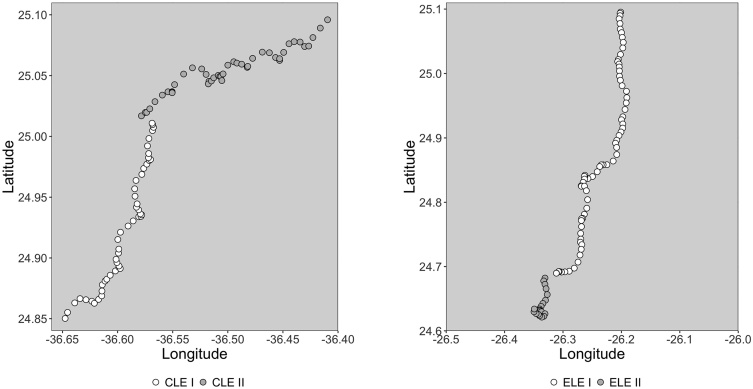
Drifter’s path during CLE I (white circles) and CLE II (grey circles) in the left panel and during ELE I (white circles) and ELE II (grey circles) in the right panel. Time between two positions is 2 h.

CLE ran for nine days, from 10/24/2006 to 11/01/2006. The drifter was recovered briefly for maintenance on 10/28/2006 and was redeployed 7 h later, 0.9 km away bearing 305° from the recovery point (Fig. 4). During this maintenance, the drogue was replaced as explained in the Supplementary Materials section. ELE went for eight days, from 11/14/2006 to 11/22/2006. Again, brief maintenance of 3 h was performed on 11/22/2006 and the drifter was redeployed 1.9 km away bearing 250° from the recovering point (Fig. 4). We will refer to these parts as CLE I and CLE II for the first and second part of the center Lagrangian experiment, and ELE I and ELE II for the first and second part of the eastern Lagrangian experiment.

We have computed the average SCADCP velocity profile for each part of each Lagrangian experiment, which is shown in Fig. 3. CLE I mean speed profile (Fig. 3a) shows a poor performance of the drogue (about 3 cm/s at the drogue depth). After the drogue and the tether cord were replaced, the performance improved significantly (Fig. 3b) and the mean slip velocities at the drogue depth decreased to about 1 cm/s during CLE II. This improvement can be credited to a larger drogue area relative to the surface float, which has been previously reported to reduce the slip velocity [5].

During ELE I the drogue showed its best performance and the mean slip at the drogue depth was less than 1 cm/s. During ELE II, the average slip value was about 1.5 cm/s.

**Fig. 5:**
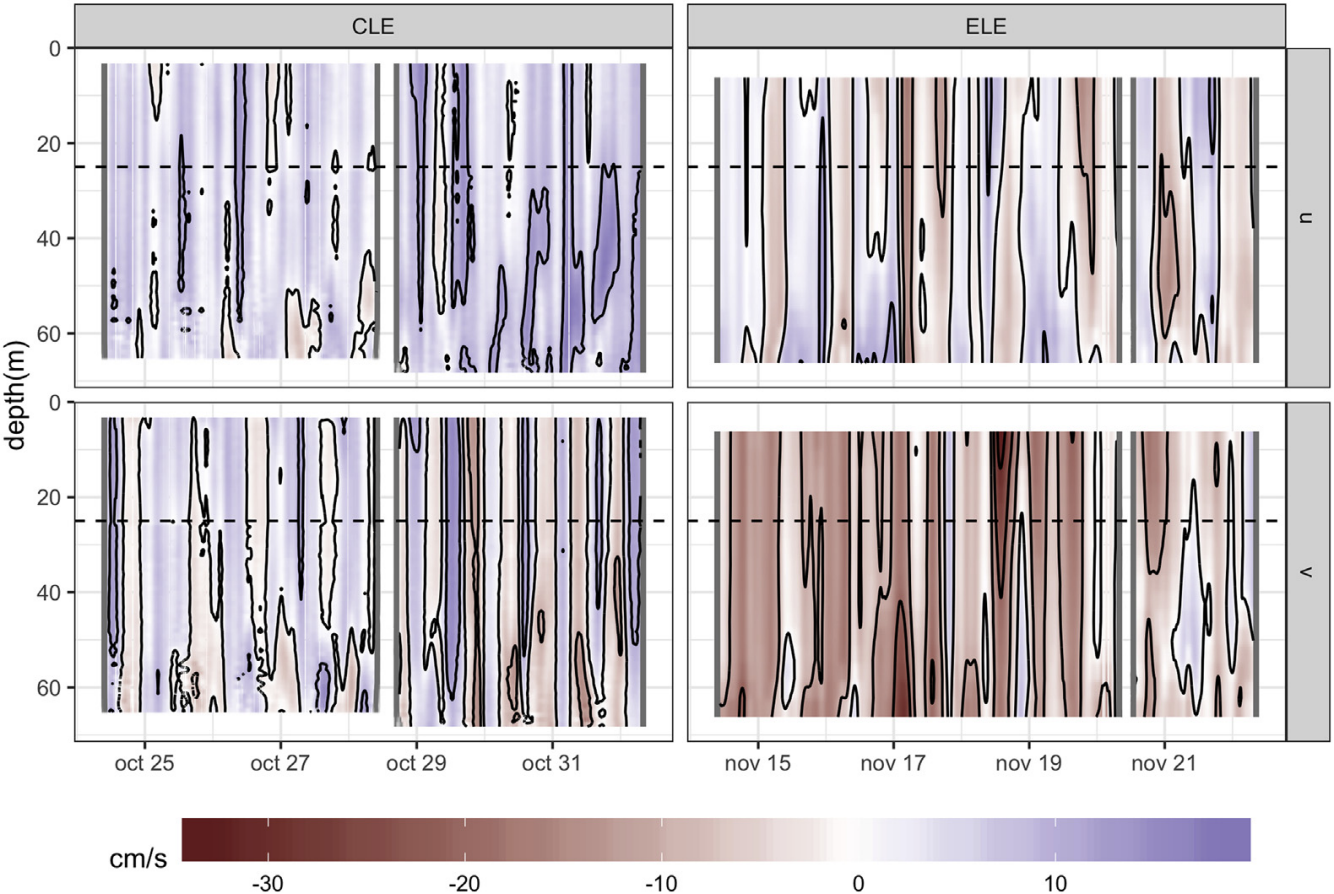
Absolute velocity profiles time series computed from Lagrangian and SCADCP data registered during CLE and ELE. U and V components shown. Solid black lines are velocity contours every 10 cm/s, dashed black lines show the theoretical position of the drogue.

Finally, as described above, a SCADCP provides with complementary data of currents at depths others than the drogue depth. We have computed the absolute velocity profiles by adding the drifter velocity estimated from GPS fixes to the relative velocity recorded at each depth of the relative velocity profiles. For this purpose, the SCADCP sampling rate should be high enough to be combined with the drifter velocity estimates from GPS fixes. Fig. 5 shows the result applied to our data.

For several days during CLE, the drogue was embedded in a layer of about 50 m depth with a weak vertical gradient. However, we can see a sharper gradient affecting the drogue by the end of the experiment. On the other hand, during ELE, the vertical shear was stronger. The wind blew stronger during the second half of both experiments. That, in turn, could have accentuated the gradient.

From the project CARPOS perspective, the combination of our slip estimation and the more or less weak vertical gradient show that, except for a couple of events, it seems reasonable to assume that, up to 50 m depth, we repeatedly sampled the same volume of water.
