# Supplementary material for: Measuring a Lagrangian drifter’s slip with an onboard ADCP
Source: MethodsX. 2019 Jun 1;6:1336–42. doi: 10.1016/j.mex.2019.05.032 (PMC6562283; doi:10.1016/j.mex.2019.05.032)
Supplement: Supplementary file 1 [file mmc1.docx]

**Additional information**

The mixed layer drifter

A surface 1.1 m diameter buoy, with a buoyancy of 276kg, was used to house satellite communications, a GPS and a Cyclops 7 fluorometer. Hanging from the buoy was a 145 m tether where a SCADCP, a drogue, and a SBE 19 CTD recorder were inserted inline. Thirteen SBE 39 temperature recorders were strapped to the tether and one SBE39 temperature and pressure recorder was fastened to the drogue. The SBE 39 were configured to sample every minute. The purpose of the SBE 39 recorders was to register the vertical distribution of the temperature during the experiments. The additional pressure sensor at the drogue was meant to check that the drogue remained at its intended depth.

A redundant satellite location system (ARGOS and Iridium) was installed in the surface buoy, and its fixes were received each hour through the Internet. Nevertheless, hourly fixes were considered not to be enough for accurate Lagrangian velocity computation and GPS fixes provided by the Iridium navigation pack were recorded every 3 seconds in a memory card housed in the buoy. The satellite navigation was also backed by a handheld GPS attached to the surface buoy. All this set of sensors attached to the buoy sum a total weight in water of approximately 20 kg.

Two window-shade type drogues were used during the experiments. First, we used a drogue 2 m high and 1.92 m wide. However, according to the slip measured during the first half of CLE, its performance was not as good as expected and was replaced during the maintenance period by a 2.5 m high, 2.40 m wide drogue. In both experiments, the drogue was located at about 25 m depth. The drogue depth was chosen after analyzing a mesoscale thermohaline field obtained before each Lagrangian experiments, and finding that the mixed layer limit was about 50 m depth.

The pressure sensor attached to the drogue measured a pressure of 27.0 db on average with a standard deviation of 0.3 db (N=5614) during CLE I and 26.8 db with a standard deviation of 0.5 db (N=5333) during CLE II. The mean value for the ELE experiment was 28.1 db with a standard deviation of 0.4 db (N=11397). These data show that the drogue remained at its intended depth.

The tether used during CLE I was a 3 mm stainless steel wire, but difficulties arisen during the deploy/recovery process advised the use of an 11 mm synthetic rope during the remaining of the experiments.
